# [Retracted] Molecular mechanisms underlying mangiferin-induced apoptosis and cell cycle arrest in A549 human lung carcinoma cells

**DOI:** 10.3892/mmr.2026.13875

**Published:** 2026-04-07

**Authors:** Wei Shi, Jiagang Deng, Rongsheng Tong, Yong Yang, Xia He, Jianzhen Lv, Hailian Wang, Shaoping Deng, Ping Qi, Dingding Zhang, Yi Wang

Mol Med Rep 13: 3423–3432, 2016; DOI: 10.3892/mmr.2016.4947

Following the publication of the above paper, it was drawn to the Editor's attention by a concerned reader that certain of the western blot data shown in Fig. 3B on p. 3428 had already appeared previously in an article written by different authors at different research institutes that was published in the journal *BMC Cancer*, but which has subsequently been retracted. Upon performing an independent analysis of the data in this paper, it also came to light that other western blot data in Figs. 4B and 5B had appeared in articles in other journals written by different authors at different research institutes, that the Control data panels in Fig. 2A and B were also overlapping within the figure itself, and other data in Fig. 2 had appeared subsequently in an unrelated paper in *Oncology Reports*.

Owing to the fact that the contentious data in the above article had already been published prior to its submission to *Molecular Medicine Reports*, the Editor has decided that this paper should be retracted from the Journal. fter having been in contact with the authors, they did not accept the decision to retract the paper. The Editor apologizes to the readership for any inconvenience caused.

